# Left hepatic trisectionectomy for hepatobiliary malignancies: Its’ role and outcomes. A retrospective cohort study

**DOI:** 10.1016/j.amsu.2019.11.016

**Published:** 2019-11-29

**Authors:** Marcos Kostalas, Adam E. Frampton, Nadeen Low, Rajiv Lahiri, Ee Jun Ban, Rajesh Kumar, Angela T. Riga, Tim R. Worthington, Nariman D. Karanjia

**Affiliations:** a HPB Surgical Unit, Royal Surrey County Hospital, Guildford, Surrey, GU2 7XX, United Kingdom; b Faculty of Health and Medical Sciences, The Leggett Building, University of Surrey, Daphne Jackson Road, Guildford, Surrey, GU2 7WG, United Kingdom; c General Surgical Unit, Wexham Park Hospital, Slough, SL2 4HL, United Kingdom; d General Surgical Unit, The Alfred Hospital, Melbourne, Victoria, 3004, Australia

**Keywords:** Left hepatic trisectionectomy, Extended left hepatectomy, Colorectal liver metastases, Hepatobiliary

## Abstract

**Background:**

Left hepatic trisectionectomy (LHT) is a complex hepatic resection; its’ role and outcomes in hepatobiliary malignancies remains unclear.

**Materials and methods:**

All patients undergoing LHT at the tertiary HPB referral unit at RSCH, Guildford, UK from September 1996 to October 2015 were included. Data were collected from a prospectively maintained database.

**Results:**

Twenty-eight patients underwent LHT. The M:F ratio was 1.8:1. Median age was 60 years (range 43–76 years). Diagnoses included colorectal liver metastases (CRLM; n = 20); cholangiocarcinoma (CCA; n = 4); and other (neuroendocrine tumour metastases (NET; n = 3) and breast metastases (n = 1)). Median duration of surgery was 270 min (range 210–585 min). Median blood loss was 750 ml (300–2400 ml) with a perioperative transfusion rate of 21% (n = 6/28). The rate of all post-operative complications was 21% for all patients, and given the extensive resection performed four patients (14%) developed varying degrees of hepatic insufficiency. One patient with cholangiocarcinoma developed severe hepatic insufficiency, which was fatal within 90 days of surgery. 1 and 3-year survivals were 92% and 68% respectively.

**Conclusion:**

This study supports LHT in patients with significant tumour burden. Despite extensive resection, our favourable morbidity and mortality rates show this is a safe and beneficial procedure for patients with all hepatobiliary malignancies. Given the nature of resection the incidence of post-operative hepatic insufficiency is higher than less extensive hepatic resections.

## Highlights

• LHT is an extended resection reported to have higher incidences of morbidity and mortality compared with less extensive hepatic resections.
• This procedure is useful for the surgical management of patients with hepatic lesions that were previously considered unresectable.
• We report favourable outcomes following LHT at our institution compared with less extensive hepatic resections.
• An initial post-operative lactate of >1.5 mmol/L was associated with an increased risk of developing post-operative complications (p = 0.035).

## 1 Introduction

Left hepatic trisectionectomy (LHT) was first described in 1982 by Starzl and colleagues [1] and later by Blumgart in 1993 [2]. The International Hepato-Pancreato-Biliary Association (IHPBA) consensus statement [3] defines the resection as excision of Couinaud segments II, III, IV, V and VIII, with or without segment I. The procedure is reserved for large left sided and central tumours that extend to involve the right anterior sectional portal pedicular structures.

Despite its’ initial description the procedure has gained slow acceptance and outcomes are limited to small case series. The first large series on peri-operative outcomes was published in 1999 ^4^, and found an increased rate of morbidity and mortality, 53% and 8% respectively, when compared with other less extensive hepatic resections. However, it highlighted the utility of LHT in lesions that were considered to previously be unresectable [4]. Other series have also demonstrated an increased morbidity and mortality rate with LHT when compared with other hepatectomies [5,6]. The increase in morbidity is attributable to the more extensive nature of the disease being treated, and the extent of hepatic resection. The procedure is therefore reserved for those with a significant tumour burden.

Long-term outcomes on patients undergoing LHT support the use of this procedure. Data regarding 1, 3 and 5-year survival has been shown to be comparable with other less extensive hepatic resections. The 1, 3 and 5 year survival data for patients with all malignancies undergoing LHT has been found to be >70%, 50% and 30% respectively in several papers [[5], [6], [7], [8]].

The aim of this study was to assess the short and long-term outcomes of LHT for patients with cholangiocarcinoma (CCA) or large volume liver metastases at our institution, and to identify any factors associated with morbidity and mortality.

## 2 Methods

All patients undergoing LHT at RSCH, Guildford, UK from September 1996 to October 2015 were included. The research registry number for the study was: researchregistry5031. Demographic data, ASA fitness grade, pre-operative intervention, neo-adjuvant chemotherapy, intra-operative and post-operative data, post-operative complications and mortality were extracted from a prospectively maintained database. Complications were graded according to the Clavien Dindo classification of surgical complications [9]. This work is fully compliant with STROCSS criteria [10]. The study is registered with the Research Registry and its unique identification number is researchregistry5031.

### 2.1 Statistical analysis

Categorical data were analysed by means of Pearson's χ2 test. The Kaplan–Meier method was used to analyse overall survival (OS). Univariate analysis was performed to show factors that had a significant influence on postoperative morbidity, 90-day mortality, and disease-specific overall survival (OS) in the univariable analysis. Date of last follow-up was December 2016. All statistical analyses were performed using SPSS® for Windows®/Mac™ version 21 (IBM, USA), and statistical significance was taken at the 5% level.

## 3 Results

During the study period, 1570 hepatic resections were performed at our HPB unit. Of these, 28 (2%) patients underwent LHT. Pre-operatively all patients were evaluated with contrast enhanced CT scan±MRI. The future liver remnant volume was assessed at our MDT and decisions were then made regarding their management. The majority of these patients had CRLM (n = 20; 72%), followed by CCA (n = 4; 14%) and then other pathologies including metastatic breast cancer, hepatocellular carcinoma and NET (n = 4; 14%). In total, there were 18 male and 10 female patients. The median age was 60 years (range 43–76 years). ASA grade was I in 6 patients (21%); II in 20 patients (71%) and III in 2 patients (7%).

Nineteen patients (68%) received neo-adjuvant chemotherapy. Of these, 17 (85%) patients had a diagnosis of CRLM, whilst 2 (50%) patients with other pathologies (metastatic breast cancer and NET) also received neo-adjuvant chemotherapy. No patients with CCA received neo-adjuvant chemotherapy treatment (Table 1). Data on the exact type of adjuvant chemotherapy after surgery was not available, as many patients return to their referring units for oncological treatment. Prior to undergoing resection, one (5%) patient with CRLM underwent pre-operative portal vein embolisation (PVE) to increase the size of future liver remnant (FLR). One patient (25%) with a diagnosis of hilar cholangiocarcinoma was found to be jaundiced pre-operatively and underwent subsequent biliary drainage with endoscopic retrograde cholangio-pancreatography (ERCP), followed by percutaneous transhepatic cholangio-pancreatography (PTC) drainage. At our institution, any patient with significant jaundice and/or cholangitis undergoes pre-operative biliary drainage.

**Table 1:** Demographics of all patients undergoing LHT according to pathology.

	Colorectal liver metastases (CRLM)	Cholangiocarcinoma (CC)	Other pathology (HCC, NET, breast)
**Number of procedures**	n = 20/28 (72%)	n = 4/28 (14%)	n = 4/28 (14%)
**M:F ratio**	15:5	1:3	2:2
**ASA grade**			
**I**	3	2	1
**II**	16	1	3
**III**	1	1	–
**Neo-adjuvant chemotherapy**	n = 17/20 (85%)	n = 0	n = 2/4 (50%)

The resections performed are shown in Table 2. Median duration of surgery was 270 min (range 210–540mins). Median blood loss in patients undergoing LHT was 750 ml (range 400–2400 ml). In total 6 patients (21%) received a peri-operative transfusion, of whom 4 (14%) received an intra-operative blood transfusion, whilst 2 (7%) patients received a blood transfusion post-operatively. The median number of units transfused in all patients undergoing LHT was 0 units (range 0–2). All six patients that required peri-operative transfusion received 2 units of packed red cells each (Table 3).

**Table 2:** Operations performed on patients with colorectal liver metastases, cholangiocarcinoma and other pathology.

	Colorectal liver metastases (CRLM)	Cholangiocarcinoma (CC)	Other pathology (HCC, NET, breast)
**L hepatectomy ext 5 + 8**	n = 13/20 (65%)	–	n = 1/4 (25%)
**L hepatectomy ext 1, 5 + 8**	n = 2/20 (10%)	n = 4/4 (100%)	n = 1/4 (25%)
**L hepatectomy ext 5 + 8 plus wedge resection**	n = 4/20 (20%)	–	n = 1/4 (25%)
**L hepatectomy ext 1, 5 + 8 plus wedge resection**	n = 1/20 (5%)	–	n = 1/4 (25%)

**Table 3:** Operative details for patients with colorectal liver metastases, cholangiocarcinoma and other pathology.

	Colorectal liver metastases (CRLM)	Cholangiocarcinoma (CC)	Other pathology (HCC, NET, breast)
**Median duration of surgery (minutes)**	270 min (range 210–480 min)	320 min (range 390–585 min)	255 min (210–540 min)
**Median blood loss (mls)**	700 ml (range 300–2400 ml)	650 ml (500–1500 ml)	1800 ml (1200–2000 ml)
**Number of patients transfused intra-operatively**	n = 3/20 (15%)	n = 2/4 (50%)	n = 1/4 (25%)
**Median number of units transfused intra-operatively**	0 (range 0–2)	0 (range 0–2)	–
**Number of patients transfused post-operatively**	n = 1/20 (5%)	–	n = 1/4 (25%)
**Median number of units transfused post-operatively**	0 (range 0–2)	–	0 (range 0–2)

The incidence of post-operative morbidity was 21% (Table 4). Six patients developed post-operative complications. Morbidity included chest infection, bile leak and post-hepatectomy liver failure. One patient developed a significant bile leak and returned to theatre for laparotomy, washout and drainage. One patient developed severe post-hepatectomy liver failure and ultimately died within 90 days. The median length of stay in hospital was 9 days (range 4–80 days).

**Table 4:** Complications of surgery classified according to Clavien-Dindo classification of surgical complications.

Clavien Dindo Grade	Number of patients (%)
**I**	n = 0 (0)
**II**	n = 1 (3.5)
**III**	n = 3 (11)
**IV**	n = 1 (3.5)
**V**	n = 1 (3.5)

**Fig. 1:**
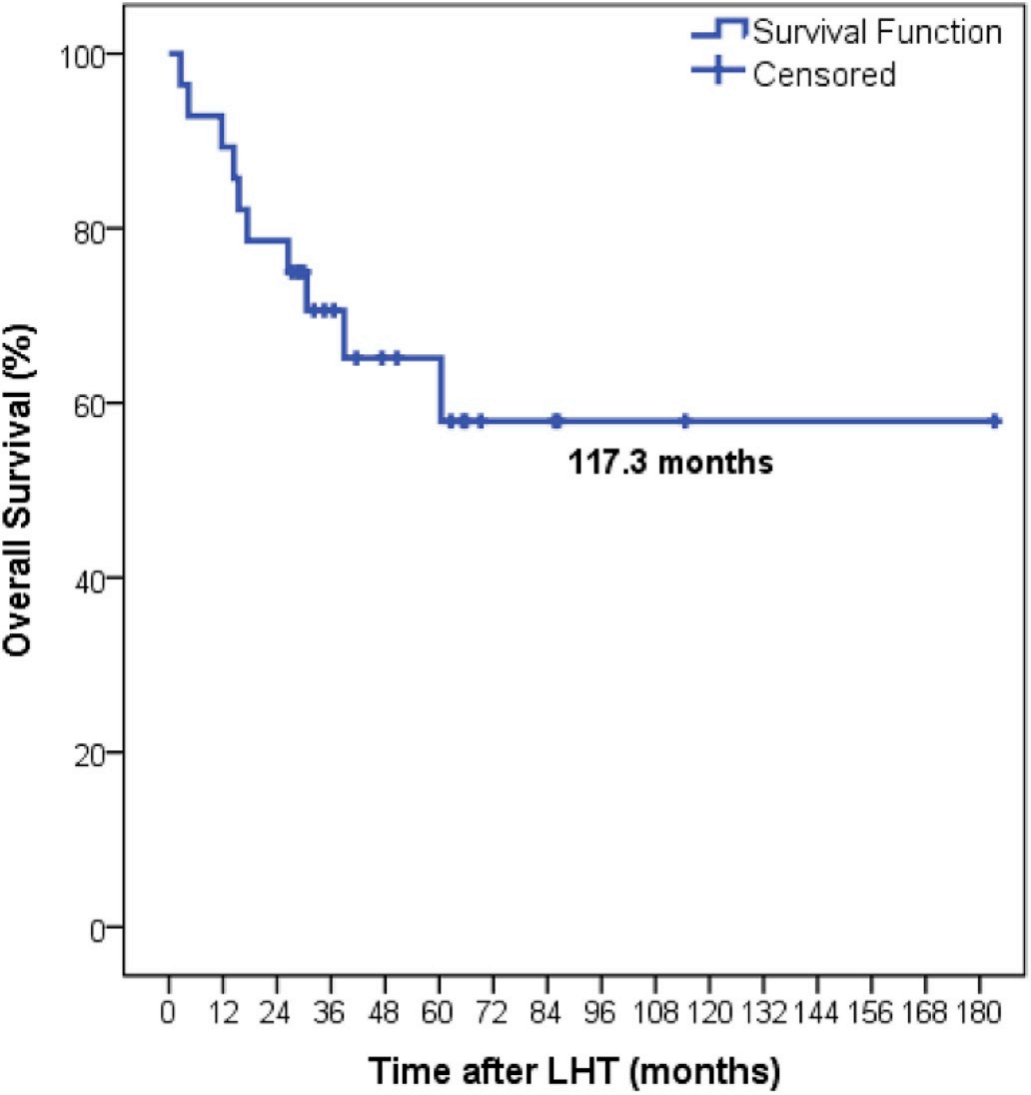
Overall survival for all patients undergoing LHT One, Three and Five-year survival rates 92%, 68% and 53% respectively with a median overall survival of 117.3 months (IQ range 84.6–149.9 months).

**Fig. 2:**
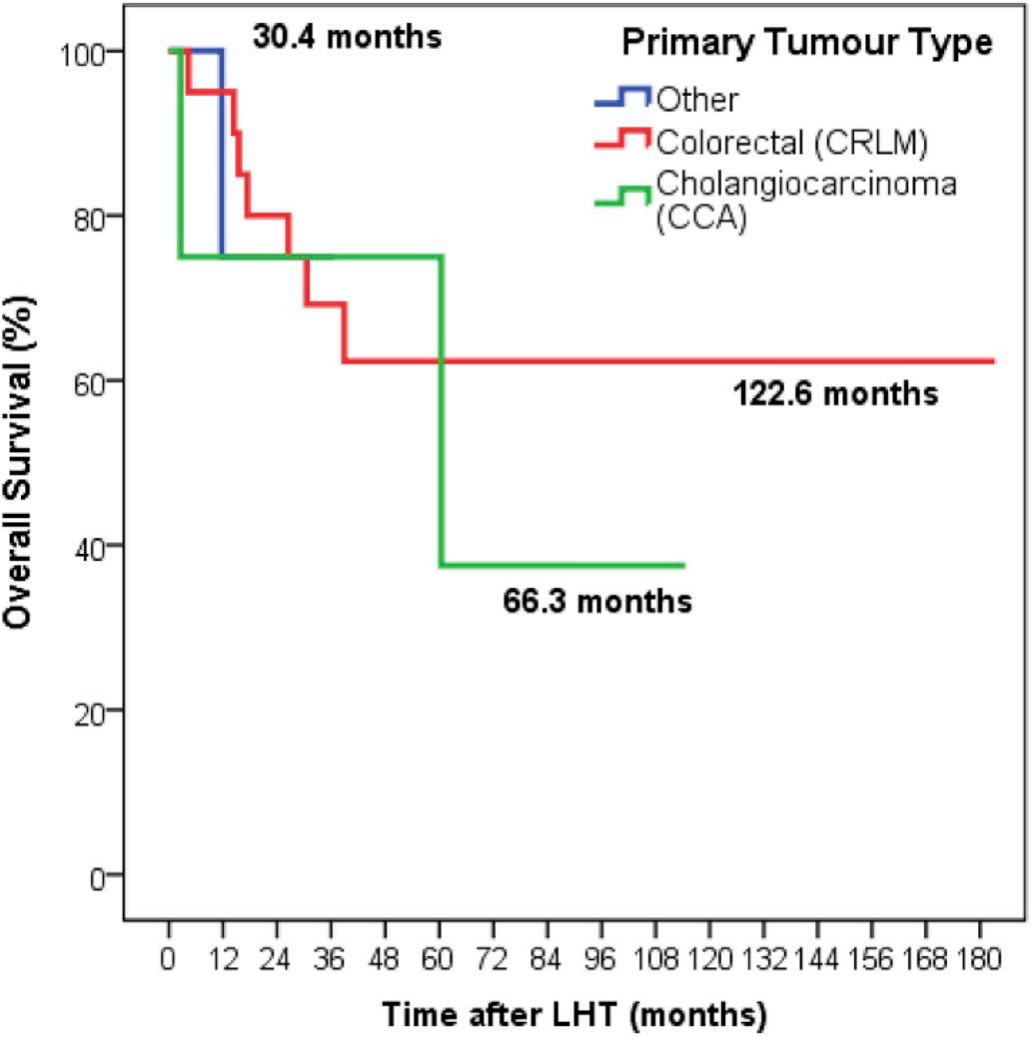
Survival curves according to tumour type (colorectal liver metastases, CRLM; cholangiocarcinoma, CC; and other pathology). Median Survival CRLM 122.6 months (IQR 86.4–158.7), CCA 66.7 months (IQR 20.5–112.0), Other 30.4 (IQR 19.8–40.9). P.

The 30-day and 90-day mortality rates for all patients undergoing LHT was 0% and 3.5% (n = 1) respectively. Survival at 1, 3 and 5 years was 92% (n = 26/28); 68% (n = 19/28); and 53% (n = 9/17) respectively. Mean OS for all patients undergoing LHT was 117 months (range 2.5–180 months; Fig. 1). By tumour type, there was no significant difference in OS between groups (Fig. 2). However, patients having LHT for CRLM tended to do better than those with CCA or other primary tumours. Table 5 shows short and long-term outcomes for patients according to their underlying pathology.

**Table 5:** Short- and long-term outcomes of patients undergoing LHT for all tumour types.

	Colorectal liver metastases (CRLAM; %)	Cholangiocarcinoma (CC; %)	Other pathology (HCC, NET, breast; %)
**30 day mortality**	n = 0/20 (0)	n = 0/4 (0)	n = 0/4 (0)
**90 day mortality**	n = 0/20 (0)	n = 1/4 (25)	n = 0/4 (0)
**1 year survival**	n = 20/20 (100)	n = 3/4 (75)	n = 3/4 (75)
**3 year survival**	n = 10/16 (62.5)	n = 3/4 (75)	n = 1/2 (50)
**5 year survival**	n = 10/15 (66)	n = 3/4 (75)	n = 0/1 (0)

Statistical analysis was performed to assess for factors associated with post-operative complications in LHT (Table 6) and OS (Table 7). Both patient and peri-operative factors were assessed. Analysis of our results found that an initial post-operative lactate of >1.5 mmol/L was associated with an increased risk of developing post-operative complications (p = 0.035). The Pringle manoeuvre was used in 9 patients undergoing LHT (32%), however this was not found to be statistically significant in the development of post-operative complications (p = 0.243). No other patient or peri-operative factors were found to be associated with increased post-operative complications, although patient age >65 just fell short of reaching statistical significance (p = 0.078). No factors were found to affect OS in our cohort (Table 7). In total, 19 patients had an R0 resection. The unit classification for an R1 resection is a margin with <1 mm clearance. There were 9 patients that were classified as having an R1 resection, whilst there were no R2 resections. As stated, resection margin status did not show any statistical significance with regards to OS.

**Table 6:** Factors associated with post-operative complications.

		Complications		Univariable
**Age (years)**	**n**	**Yes**	**No**	*P* = 0.078
<65	15	6	10	
>65	13	1	12	

**Sex**	n			*P* = 0.172
M	18	6	12	
F	10	1	9	

**ASA**				*P* = 0.397
I	6	1	5	
II	20	6	15	
III	2	0	2	

**Primary Tumour**				*P* = 0.449
CRLM	20	4	16	
Cholangiocarcinoma	4	0	4	
Other	4	1	3	

**Adjuvant Chemotherapy**				*P* = 0.663
Yes	14	4	10	
No	14	3	11	

**Neo-adjuvant Chemotherapy**				*P* = 0.815
Yes	19	5	14	
No	9	2	7	

**Jaundice**				*P* = 0.563
Yes	2	1	1	
No	26	5	21	

**Duration of surgery**				*P* = 0.577
270 min	9	2	7	
>270 min	13	4	6	

**Intraoperative blood loss >1L**				*P* = 0.503
Blood loss <1L	17	5	12	
Blood loss > 1L	11	2	9	

**Blood transfusion**				*P* = 0.595
Yes	6	1	5	
No	22	6	16	

**Segment 1 resection**				*P* = 0.172
Yes	10	4	6	
No	18	3	15	

**Segment VI/VII metastasectomy**				*P* = 0.801
Yes	7	2	5	
No	21	5	16	

**R1/0 resection**				*P* = 0.483
R1	9	3	6	
R0	19	4	15	

**Pringle manoeuvre**				*P* = 0.243
Yes	9	1	8	
No	19	6	13	

**Pringle manoeuvre duration**				*P* = 0.595
<10 min	22	6	16	
>10 min	6	1	5	

**Post-operative lactate**				***P* = 0.035**
Lactate >1.5	19	7	12	
Lactate <1.5	9	0	9

**Table 7:** Factors associated with overall survival.

		Median survival (months)	Range (months)	Univariable
**Age (years)**	**n**			*P* = 0.216
<65	16	131.7	87.1–176.3	
>65	12	63.8	35.4–92.2	

**Sex**	n			*P* = 0.958
M	18	123.8	84.8–162.8	
F	10	78.2	50.7–105.7	

**ASA**				*P* = 0.553
I	6	47.6	29.6–65.5	
II–III	22	124.5	88.6–160	

**Adjuvant Chemotherapy**				*P* = 0.411
Yes	14	135.1	95.1–107.1	
No	14	63.9	36.5–91.2	

**Neo-adjuvant chemotherapy**				*P* = 0.602
Yes	19	130.1	94.9–165.4	
No	9	61.6	30.0–93.1	

**Post-op Complications**				P = 0.998
Yes	7	63.8	37.2–90.3	
No	21	115.8	79.7–152.1	

**R0/R1 resection**				P = 0.267
R0 resection	19	102.4	60.3–144.5	
R1 resection	9	71.4	53.2–89.7	

**Duration of surgery**				P = 0.572
270 min	9	54.1	39.5–68.6	
>270 min	13	61.7	41.8–81.7	

Blood loss				P = 0.282
<1L	17	129.7	90.5–169.0	
>1L	11	50.2	27.8–72.7	

**Blood transfusion**				P = 0.504
Yes	6	45.7	27.6–63.9	
No	22	125.7	90.3–161.1	

**Segment 1 resection**				P = 0.219
Yes	10	91.2	63.6–118.8	
No	18	104.6	64.2–145.0	

**Segment VI/VII metastasectomy**				P = 0.244
Yes	7	75.9	58.2–93.6	
No	21	106.1	68.7–143.7	

**Pringle manoeuvre**				P = 0.262
Yes	9	145.8	100–191.6	
No	19	55.0	39.1–70.9	

**Duration of Pringle manouevre**				P = 0.299
<10 min	22	105.4	66.9–144.0	
>10 min	6	97.5	84.7–149.9	

**Post-operative lactate**				P = 0.246
<1.5	9	92.6	65.4–119.7	
>1.5	19	95.9	51.5–140.4	

## 4 Discussion

In this case series, we reviewed the short and long-term outcomes of patients undergoing LHT at RSCH, Guildford, UK. Previous published data has identified pre-operative jaundice and intraoperative blood transfusion as independent predictors of post-operative morbidity [6,11,12]. We did not find pre-operative jaundice to be a predictor, but only 2 patients were jaundiced and both were stented prior to LHT. Data from our series identified a raised post-operative lactate of >1.5 as a predictor for post-operative complications (p = 0.035). No other factors were identified.

The overall incidence of post-operative complications after LHT was 21% (n = 6) and is low, especially given the extent of hepatic resection undertaken during the procedure. Other series of LHT have quoted complication rates of more than 45% [5,6,8]. These included transient hepatic insufficiency, bile leak and post-operative chest infection. Only one of our patients required return to theatre, for laparotomy and washout due to a bile leak. Only one patient in our series required pre-operative PVE, whilst two patients underwent pre-operative biliary drainage by PTC (with one having attempted drainage with ERCP in the first instance) prior to LHT. The majority of patients receiving neo-adjuvant chemotherapy were those with CRLM, whilst two patients with other pathology (metastatic NET and metastatic breast cancer) also received pre-operative chemotherapy. There was no consensus on the type of chemotherapy regimen received.

Overall 30 and 90-day mortalities of zero and 3.5% are also well within rates of 7% and 9% quoted by other series [6,8]. One, three and five-year survival rates of 92%, 68% and 53% are also very reassuring, and are better than other series [5,6,13,14], further supporting the safety of the procedure and its utilisation in the management of patients with a large tumour burden. The median OS for all patients undergoing LHT at our centre was 117.3 months (range 2.5–180 months). The median OS for patients with CRLM was 122.6 months (range 86.4–158.7 months). Although OS rates were improved in this group, compared to those undergoing LHT for CCA and other tumours, it did not reach statistical significance. This data compares favourably to other series of LHT for CRLM [15] and adds further weight to evidence that LHT can provide an excellent long-term survival in these patients.

There was one patient death within 90 days of surgery. This patient had an underlying diagnosis of CCA and underwent LHT, including resection of the caudate lobe. Post-operatively the patient had persistently elevated liver function tests with bilirubin >300 μmol/L and ALP >400 IU /L for more than five days post-operatively and required multi-organ support in the intensive care unit. The patient was referred for porto-caval shunting due to “small for size syndrome” and liver insufficiency at a neighbouring institution. However, the patient developed vasopressor resistant sepsis and died shortly after this.

One of the major risks associated with hepatic resection is blood loss [6,11,12]. Dionigi et al. [16] found an increased rate of complications in patients undergoing hepatic resection that required peri-operative transfusion. The median blood loss in our series was 750 ml (range 300–2400 ml) and 6 patients (21%) required peri-operative transfusion. Of these, one patient experienced a bile leak post-operatively and this was managed conservatively by radiological drain insertion. We found that peri-operative transfusion was not associated with a reduction in OS. However, our findings regarding peri-operative transfusion are limited by the relatively small sample size of this cohort. Our data for blood loss is lower than that quoted in a study by Zhou et al., in which the mean blood loss in patients undergoing trisectionectomy was 1351 ml [17]. Our results suggest a favourable surgical and anaesthetic technique. Notably, the threshold for transfusion was haemoglobin <7.5 g/dL, or <10 g/dL in patients with coronary artery disease or symptomatic anaemia. Despite such an extensive resection, very few patients required a blood transfusion and this may account for the low rate of complications and improved survival outcomes compared to other LHT series.

The data from our series would suggest that despite undergoing such an extensive resection, LHT is a relatively safe procedure and provides excellent survival outcomes for patients with CRLM.

### 4.1 Limitations

The study is limited by its small sample size, and therefore the lack of predictors for post-operative morbidity and mortality may be a Type II statistical error.

## 5 Conclusions

In this small case series, we show that LHT is an effective treatment in patients with CRLM and significant tumour burden offering excellent OS.

## Provenance and peer review

Not commissioned, externally peer reviewed.

## Ethical approval

Not required.

## Funding sources

None to declare.

## Financial disclosure

None reported.

## Contributions

***Study concept and design***: Kostalas, Frampton, Karanjia.

***Acquisition of data***: Kostalas, Frampton, Kumar, Riga, Worthington, Karanjia.

***Analysis and interpretation of data***: Kostalas, Frampton, Karanjia.

***Drafting of the manuscript***: Kostalas, Frampton, Karanjia.

***Critical revision of the manuscript for important intellectual content***: Kostalas, Frampton, Lahiri, Ban, Low, Karanjia.

***Study supervision***: Karanjia.

## Registration of research studies

In accordance with the Declaration of Helsinki 2013, all research involving human participants has to be registered in a publicly accessible database. Please enter the name of the registry and the unique identifying number (UIN) of your study.

You can register any type of research at http://www.researchregistry.com to obtain your UIN if you have not already registered. This is mandatory for human studies only.  Trials and certain observational research can also be registered elsewhere such as: ClinicalTrials.gov or ISRCTN or numerous other registries.

## Guarantor

The Guarantor is the one or more people who accept full responsibility for the work and/or the conduct of the study, had access to the data, and controlled the decision to publish

## Declaration of competing interest

There are no conflicts of interest to declare.

## References

[1] Starzl T.E., Iwatsuki S., Shaw B.W. (1982). Left hepatic trisegmentectomy. Surg. Gynecol. Obstet..

[2] Blumgart L.H., Baer H.U., Czerniak A., Zimmermann A., Dennison A.R. (1993). Extended left hepatectomy: technical aspects of an evolving procedure. Br. J. Surg..

[3] Pang Y.Y. (2002). The Brisbane 2000 terminology of liver anatomy and resections. HPB 2000; 2:333-339. HPB : Off. J. HPB.

[4] Povoski S.P., Fong Y., Blumgart L.H. (1999). Extended left hepatectomy. World J. Surg..

[5] Lang H., Sotiropoulos G.C., Brokalaki E.I. (2006). Left hepatic trisectionectomy for hepatobiliary malignancies. J. Am. Coll. Surg..

[6] Farid S.G., White A., Khan N., Toogood G.J., Prasad K.R., Lodge J.P. (2016). Clinical outcomes of left hepatic trisectionectomy for hepatobiliary malignancy. Br. J. Surg..

[7] Vauthey J.N., Pawlik T.M., Abdalla E.K. (2004). Is extended hepatectomy for hepatobiliary malignancy justified?. Ann. Surg..

[8] Nishio H., Hidalgo E., Hamady Z.Z. (2005). Left hepatic trisectionectomy for hepatobiliary malignancy: results and an appraisal of its current role. Ann. Surg..

[9] Dindo D., Demartines N., Clavien P.A. (2004). Classification of surgical complications: a new proposal with evaluation in a cohort of 6336 patients and results of a survey. Ann. Surg..

[10] Agha R.A., Borrelli M.R., Vella-Baldacchino M., Thavayogan R., Orgill D.P. (2017). The STROCSS statement: strengthening the reporting of cohort studies in surgery. Int. J. Surg..

[11] Kooby D.A., Stockman J., Ben-Porat L. (2003). Influence of transfusions on perioperative and long-term outcome in patients following hepatic resection for colorectal metastases. Ann. Surg..

[12] Schiergens T.S., Rentsch M., Kasparek M.S., Frenes K., Jauch K.W., Thasler W.E. (2015). Impact of perioperative allogeneic red blood cell transfusion on recurrence and overall survival after resection of colorectal liver metastases. Dis. Colon Rectum.

[13] Hidalgo E., Asthana S., Nishio H. (2008). Surgery for hilar cholangiocarcinoma: the Leeds experience. Eur. J. Surg. Oncol: J. Eur. Soc. Surg. Oncol. Br. Assoc. Surg. Oncol..

[14] Hemming A.W., Reed A.I., Langham M.R., Fujita S., Howard R.J. (2004). Combined resection of the liver and inferior vena cava for hepatic malignancy. Ann. Surg..

[15] Wicherts D.A., de Haas R.J., Andreani P. (2011). Short- and long-term results of extended left hepatectomy for colorectal metastases. HPB.

[16] Dionigi G., Boni L., Rovera F. (2009). Effect of perioperative blood transfusion on clinical outcomes in hepatic surgery for cancer. World J. Gastroenterol..

[17] Zhou Y., Sui C., Li B., Kan T., Yang J., Wu M. (2011). Safety and efficacy of trisectionectomy for hepatocellular carcinoma. ANZ J. Surg..

